# Development and validation of multidimensional nomograms for predicting prostate cancer risk: a retrospective study

**DOI:** 10.3389/fonc.2026.1883224

**Published:** 2026-06-30

**Authors:** Tao Zhang, Xue Li, Junsong Zeng, Maosen Xu, Yan Tie

**Affiliations:** 1 Department of Biotherapy, Cancer Center and State Key Laboratory of Biotherapy, West China Hospital, Sichuan University, Chengdu, China; 2 West China School of Medicine and State Key Laboratory of Biotherapy, West China Hospital, Sichuan University, Chengdu, China; 3 Cancer Center and State Key Laboratory of Biotherapy, West China Hospital, Sichuan University, Chengdu, China

**Keywords:** diagnosis, metabolic markers, nomogram, pathological grade, prostate cancer, risk stratification, TyG index

## Abstract

**Background:**

PSA screening has limited specificity in the 10–20 ng/mL range, making biopsy decisions difficult. Metabolic disorders and inflammation may improve risk prediction. We aimed to develop a model integrating multidimensional indicators to predict prostate cancer and high−grade disease in men with tPSA >10 ng/mL.

**Methods:**

This retrospective study included 461 men who underwent prostate biopsy (tPSA >10 ng/mL). Patients were randomly split into training (70%) and validation (30%) sets. Two logistic regression models were built: Model 1 for benign vs. malignant; Model 2 (in malignant cases) for high−grade (ISUP ≥3) vs. low−grade. Variables with VIF>5 were excluded. Backward stepwise (AIC) and univariate *P* < 0.05 guided variable selection. Performance was assessed by AUC, calibration, DCA, and compared with fPSA% alone using DeLong test.

**Result:**

Among 461 patients, 252 (54.7%) had prostate cancer, and 139 (55.2% of malignant cases) had high−grade disease. Malignant patients were older, had higher BMI, TyG index, NLR, and tPSA, and lower fPSA% (all *P* < 0.05). High−grade patients showed similar metabolic differences but no tPSA difference versus low−grade. Model 1 retained age, TyG index, NLR, fPSA%, smoking, hypertension, and lesion location. Model 2 retained age, TyG index, LDH, and smoking history. Validation AUC was 0.871 for Model 1 and 0.779 for Model 2. At optimal thresholds, sensitivity/specificity were 80.3%/74.6% (Model 1) and 71.7%/62.1% (Model 2). Both models showed good calibration (Hosmer−Lemeshow P>0.05). Decision curve analysis and DeLong test confirmed that each full model provided higher net benefit than fPSA% alone (*P* < 0.05).

**Conclusion:**

Two nomograms using routine clinical and laboratory variables may assist in risk stratification for prostate cancer and high−grade disease in men with tPSA >10 ng/mL, outperforming fPSA% alone. These tools can help reduce unnecessary biopsies.

## Introduction

Prostate cancer is a highly prevalent malignant tumor unique to men and one of the main causes of cancer-related deaths in men (1). With population aging and the widespread use of prostate-specific antigen (PSA) screening, the detection rate of prostate cancer has increased significantly.

PSA is the most widely used initial screening laboratory indicator for prostate cancer (2). Current clinical guidelines generally use total PSA level and free PSA percentage as important reference indicators for evaluating prostate cancer risk and recurrence monitoring (3–5). However, benign lesions and inflammation can also cause elevated PSA, which results in low specificity. Especially in the “intermediate group” with moderately elevated PSA (10–20 ng/mL), it’s difficult to evaluate malignancy solely based on PSA levels as an evaluation indicator (3). To ensure accurate diagnosis, doctors usually require additional imaging examinations and pathological biopsy results to assist in judgment. So, it has important clinical value to develop an accurate and non-invasive predictive tool for patients, which can reduce pain caused by biopsy and waste of medical resources.

In recent years, the relationship between metabolic disorder, inflammation, and prostate cancer has received increasing attention (6–8). Regarding metabolism, components of metabolic syndrome such as obesity, insulin resistance, and lipid disorder have been shown to promote the occurrence and progression of prostate cancer (9–12). Obesity is a commonly used nutritional and metabolic risk factor for prostate cancer which had been clearly recognized. Obesity creates a favorable internal environment for the occurrence and development of prostate cancer by promoting chronic inflammation, insulin resistance, and hormonal metabolism disorders (13, 14). Systemic glucose metabolism disorders have also been found to promote tumor occurrence and development (15, 16). Among all metabolic indicators, the triglyceride-glucose (TyG) index is a widely used prospective diagnostic indicator for insulin resistance and diabetes, and it has been confirmed to be associated with the risk of prostate cancer (17–19).

In addition to glucose metabolism, abnormal lipid metabolism can also lead to the occurrence of prostate cancer (20, 21). Lipids not only maintain the cell membrane structure, provide energy, regulate redox homeostasis, but also promote plasticity, angiogenesis, and microenvironment remodeling, mediate inflammatory responses, and participate widely as signaling molecules in various processes of tumor development (22–24). Dysregulation of lipid metabolism not only disrupts the physiological functions of normal cells, but also promotes the generation and survival of cancer cells (25).

Inflammation, as one of the Hallmarks of tumors, also plays an important role in the development of prostate cancer (26–28). For example, the neutrophil to lymphocyte ratio (NLR), as an inexpensive and easily obtainable inflammatory marker, has been reported to be associated with adverse pathological features and survival prognosis of prostate cancer (29–31).

Although some studies have separately explored the relationship between metabolic or inflammatory indicators and prostate cancer risk, most of these studies have small sample sizes, or only explore whether a single indicator is a risk factor for tumor occurrence (32–34). So far, no study has systematically integrated metabolic, inflammatory, and routine laboratory indicators together to analyze and construct a comprehensive model for predicting benign versus malignant prostate lesions in the high PSA population.

Based on the above background, this study retrospectively collected patients who have been pathologically confirmed by prostate biopsy. We attempt to construct predictive models to distinguish benign lesions from prostate cancer, and within malignant patients, distinguish high-grade from low-grade cancer. These multidimensional indicators include demographic characteristics, metabolic indicators, inflammatory indicators, PSA levels, and other common laboratory test indicators.

This study aims to construct a diagnostic model in the high PSA level population, provide reliable diagnostic information for clinical decision-making. It can reduce the additional economic costs and additional trauma from extra imaging examinations and pathological biopsy.

## Methods

### Study population

This study is a single-center, retrospective observational study. Male patients who underwent prostate biopsy at West China Hospital from May 2021 to May 2024 were enrolled.

All patients met the following inclusion criteria: first prostate biopsy at our center; serum total PSA (tPSA) > 10 ng/mL measured at our center within one month prior to biopsy; complete clinical, laboratory, and pathological data available at our center. Exclusion criteria included: prior diagnosis or treatment for prostate cancer; presence of other malignancies; acute prostatitis within 2 months before biopsy. Acute prostatitis was defined on the basis of clinical symptoms or laboratory abnormalities as recorded in the electronic medical records.

### Data collection

We collected baseline information from all participants in the electronic medical record system, and all laboratory test samples were collected by professional doctors or nurses at our center and analyzed and processed according to standardized procedures.

In this study, the patient data we collected included the following categories: (1) demographic characteristics, lifestyle, and history of chronic diseases; (2) serum metabolic indicators; (3) blood routine indicators; (4) liver and kidney function and serum enzymology; (5) prostate specific antigen and its derived indicators; (6) prostate lesion location and pathological biopsy results. Lesion location (peripheral zone, transition zone, or central zone) was determined by pre-biopsy MRI or ultrasound. TyG index was calculated as ln[fasting TG (mg/dL)*fasting glucose (mg/dL)/2]. TG and glucose levels were first converted from mmol/L to mg/dL using standard conversion factors (TG:*88.57, glucose:*18.0).

### Statistical analysis

Because all enrolled patients underwent routine clinical and laboratory assessments at our center, and our inclusion criteria required complete data for each participant, there were no missing values for any of the variables included in the final analysis. Consequently, no imputation was needed. We used a stratified random sampling method to divide all patients into a training set and a validation set in a 7:3 ratio. The stratification was based on pathological diagnosis results (benign/malignant) to ensure consistent proportions of benign and malignant in both groups. To ensure the reproducibility of the results, we also set up random seeds. To avoid the impact of data collinearity, we used variance inflation factor (VIF) to diagnose whether the data have multicollinearity and excluded variables with VIF>5.

In this study, we constructed two prediction models for two distinct endpoints: differentiation between benign and malignant lesions (based on pathological results) and differentiation between pathological high-grade and low-grade lesions among malignant cases (based on ISUP grade). For the first model, which aimed to distinguish benign from malignant lesions, we used pathological outcome (benign vs. malignant) as the dependent variable and built a logistic regression model using the training set. For the second model, which addressed pathological grading, we included only patients with malignant lesions, took high-grade (high-grade vs. low-grade) as the dependent variable, and developed a logistic regression model on the training set. For Model 2, the same 7:3 stratified split was applied exclusively within the malignant subgroup; consequently, the validation sets for the two models are not identical (Model 1 validation includes benign patients, whereas Model 2 validation includes only malignant patients). In both models, we first performed univariate logistic regression to select variables with *P* < 0.1; we then incorporated these variables into multivariate models and applied backward stepwise regression to select the optimal combination of predictors. Based on the final results of the two models, we also plotted nomograms to visualize individual predicted probabilities.

We evaluated the performance of models from two dimensions, discrimination and calibration. To compare with the clinically commonly used percentage of free PSA (fPSA%), we applied the DeLong test to evaluate the difference in the area under the curve (AUC) between each of our two discrimination models and the fPSA% alone in predicting pathological outcomes. We also plotted receiver operating characteristic (ROC) curves, and calculated the optimal thresholds (Youden index), sensitivity, specificity, positive predictive value (PPV) and negative predictive value (NPV) in both the training and validation sets. For internal validation, we performed bootstrap resampling with 200 replicates on the two models and computed the C-index. To assess model calibration, we plotted calibration curves in both the training and validation sets to evaluate model fit.

To evaluate the clinical efficacy of the two models, we used decision curve analysis (DCA) to assess the net benefits of our model relative to the “full intervention” and “no intervention” strategies in the validation set, and compared it with the strategy using a single fPSA% indicator.

All statistical analyses were conducted using R (version 4.6.0). Continuous variables are represented by mean ± SD, while categorical variables are represented by frequency (percentage). The Mann Whitney U test or chi square test was used for inter group comparison. Two-sided *P* < 0.05 is considered statistically significant.

## Results

### Clinical characteristics of the enrolled population

According to the inclusion criteria, 2186 patients were initially screened in this study. 1331 patients with previous diagnosis or treatment of prostate cancer, 142 patients with other malignant tumors, and 252 patients with a history of non-tumor prostate diseases such as acute prostatitis within 2 months before puncture were excluded In the end, a total of 461 patients were included in this study,. Among them, there were 209 cases (45.3%) of benign lesions and 252 cases (54.7%) of malignant lesions.

Compared with benign patients, malignant patients are older (*P* = 0.012) and have a higher BMI (*P* = 0.004). In addition, malignant patients had higher Total PSA levels (*P* < 0.001), but lower Free PSA and Free PSA% (*P* < 0.001), and significantly increased NLR and TyG index (both *P* < 0.05). The differences in detailed baseline information between the two groups are detailed in **Table 1**.

**Table 1:** Baseline characteristics of the study population (benign vs. malignant).

Variables	Benign group (n=209)	Malignant group (n=252)	*P*
Age (years, mean ± SD)	67.0 (8.1)	72.4 (7.6)	**0.012**
BMI (Kg/m2, mean ± SD)	23.51 (3.25)	24.48 (3.83)	**0.004**
Smoking history (%)			<0.001
Yes	88 (42.1)	196 (77.8)	
No	121(57.9)	56 (22.2)	
Hypertension (%)			0.042
Yes	77 (36.8)	148 (58.7)	
No	132 (63.2)	104 (41.3)	
Triglycerides (mmol/L, mean ± SD)	1.55 (0.30)	1.97 (0.57)	**<0.001**
Plasma Glucose (mmol/L, mean ± SD)	5.84 (1.30)	6.55 (1.48)	**<0.001**
TyG Index(mmol/L, mean ± SD)	8.84 (0.29)	9.16 (0.45)	**<0.001**
Total Cholesterol (mmol/L, mean ± SD)	4.60 (0.70)	4.80 (0.68)	**0.002**
Low−Density Lipoprotein (mmol/L, mean ± SD)	2.67 (0.65)	2.70 (0.63)	0.687
High−Density Lipoprotein (mmol/L, mean ± SD)	1.22 (0.28)	1.21 (0.28)	0.584
Red Blood Cell Count (× 1012/L, mean ± SD)	4.65 (0.45)	4.59 (0.44)	0.149
Hemoglobin (g/L, mean ± SD)	139.97 (13.42)	137.99 (12.95)	0.109
Platelets (× 109/L, mean ± SD)	223.35 (29.40)	226.90 (30.34)	0.205
White Blood Cell Count (× 109/L, mean ± SD)	7.55 (3.33)	7.99 (3.44)	0.166
Neutrophils (× 109/L, mean ± SD)	4.45 (2.16)	5.05 (2.36)	**0.005**
Lymphocytes (× 109/L), mean ± SD)	2.17 (1.00)	2.14 (1.08)	0.739
Neutrophil/Lymphocyte Ratio (mean ± SD)	2.11 (0.63)	2.67 (0.87)	**0.009**
Alanine Aminotransferase (U/L, mean ± SD)	29.15 (17.40)	26.48 (16.18)	0.089
Aspartate Aminotransferase (U/L, mean ± SD)	40.26 (23.85)	36.58 (22.38)	0.104
Alkaline Phosphatase (U/L, mean ± SD)	132.34 (79.07)	119.99 (74.93)	0.116
Lactate Dehydrogenase (U/L, mean ± SD)	170.26 (18.25)	183.23 (30.48)	**<0.001**
Total PSA (ng/dL, mean ± SD)	14.78 (2.88)	16.84 (5.09)	**0.007**
Free PSA (ng/dL, mean ± SD)	2.87 (1.34)	2.18 (1.19)	**<0.001**
Free PSA% (mean ± SD)	19.31 (7.60)	14.38 (8.37)	**<0.001**
Lesion location (%)			<0.001
Peripheral Zone	59 (28.2)	169 (67.1)	
Transitional Zone	138 (66.0)	75 (29.8)	
Central Zone	12 (5.7)	8 (3.2)	

Bold values indicate statistical significance. SD, Standard Deviation; PSA, prostate-specific antigen.

Among all malignant patients, there were 139 cases (55.2%) diagnosed with high-grade cancer (ISUP grades 3, 4, and 5) and 113 cases (44.8%) diagnosed with low-grade cancer (ISUP grades 1 and 2). Similar to the differences between benign and malignant patients, high-grade cancer patients are older, have higher BMI and TyG index, and lower fPSA% compared to low-grade cancer patients (all *P* < 0.05). It is worth noting that although there are differences in total PSA levels and lesion location in the comparison of benign and malignant patient groups, no differences were shown in the comparison of high and low pathological grade groups in malignant patients (*P*>0.05). The baseline characteristics comparison between high-grade cancer and low-grade cancer is shown in **Table 2**.

**Table 2:** Baseline characteristics of malignant patients (high-grade vs. low-grade).

Variables	High-grade (n=139)	Low-grade (n=113)	*P*
Age (years, mean ± SD)	74.1 (7.1)	70.4 (7.6.7)	**<0.001**
BMI (Kg/m2, mean ± SD)	24.87 (3.73)	23.99 (3.92)	**0.069**
Smoking history (%)			<0.001
Yes	122 (87.8)	74 (65.5)	
No	17 (12.2)	39 (34.5)	
Hypertension (%)			0.131
Yes	88 (63.3)	60 (53.1)	
No	51 (36.7)	53 (46.9)	
Triglycerides (mmol/L, mean ± SD)	2.21 (0.58)	1.69 (0.40)	**<0.001**
Plasma Glucose (mmol/L, mean ± SD)	6.49 (1.44)	6.64 (1.52)	0.424
TyG Index(mmol/L, mean ± SD)	9.26 (0.51)	9.04 (0.34)	**<0.001**
Total Cholesterol (mmol/L, mean ± SD)	4.89 (0.72)	4.69 (0.62)	**0.017**
Low-Density Lipoprotein (mmol/L, mean ± SD)	2.69 (0.66)	2.70 (0.59)	0.872
High-Density Lipoprotein (mmol/L, mean ± SD)	1.20 (0.28)	1.22 (0.29)	0.630
Red Blood Cell Count (× 1012/L, mean ± SD)	4.59 (0.45)	4.59 (0.44)	0.983
Hemoglobin (g/L, mean ± SD)	137.99 (13.07)	137.99 (12.86)	0.997
Platelets (× 109/L, mean ± SD)	230.78 (29.55)	222.14 (30.74)	**0.024**
White Blood Cell Count (× 109/L, mean ± SD)	8.11 (3.57)	7.84 (3.27)	0.536
Neutrophils (× 109/L, mean ± SD)	5.20 (2.47)	4.87 (2.22)	0.276
Lymphocytes (× 109/L), mean ± SD)	2.09 (1.06)	2.20 (1.10)	0.405
Neutrophil/Lymphocyte Ratio (mean ± SD)	2.80 (0.90)	2.51 (0.81)	**0.009**
Alanine Aminotransferase (U/L, mean ± SD)	25.52 (15.82)	27.65 (16.61)	0.298
Aspartate Aminotransferase (U/L, mean ± SD)	35.11 (21.66)	38.39 (23.22)	0.249
Alkaline Phosphatase (U/L, mean ± SD)	115.04 (71.62)	126.06 (78.71)	0.246
Lactate Dehydrogenase (U/L, mean ± SD)	190.11 (30.83)	174.76 (27.92)	**<0.001**
Total PSA (ng/dL, mean ± SD)	17.04 (4.97)	16.60 (5.24)	0.493
Free PSA (ng/dL, mean ± SD)	2.11 (1.30)	2.27 (1.04)	0.289
Free PSA% (mean ± SD)	13.04 (7.18)	16.04 (9.40)	**0.005**
Lesion location (%)			0.113
Peripheral Zone	99 (71.2)	70 (61.9)	
Transitional Zone	38 (27.3)	37 (32.7)	
Central Zone	2 (1.4)	6 (5.3)	

Bold values indicate statistical significance. SD, Standard Deviation; PSA, prostate-specific antigen.

### Variable selection and logistic regression analysis

We divided all patients into a training set (n=322) and a validation set (n=139) in a 7:3 ratio, and the model construction was based on the training set data. We conducted collinearity diagnostic analysis on all variables before constructing the models. By calculating the VIF of each variable, we excluded variables with VIF>5, including plasma glucose, total cholesterol, LDL, RBC count, hemoglobin, neutrophils, lymphocytes, ALT, AST, ALP and free PSA. The continuous variables ultimately included in the univariate analysis include: age, BMI, TyG index, HDL, platelets, WBC count, NLR, LDH, total PSA, free PSA% and categorical variables: smoking history, hypertension, and lesion location. The VIF values of all variables are shown in **Supplementary Table 1**. Because absolute fPSA was highly correlated with tPSA (VIF 11.15), we excluded it to avoid multicollinearity. However, fPSA% (fPSA/tPSA) provides complementary clinical information and was retained in the analysis after confirming that its VIF was below 5.

The detailed results of univariate logistic regression for all variables are provided in **Supplementary Table 2**. In the model used to distinguish benign and malignant prostate lesions (hereinafter referred to as Model 1), the results of univariate analysis suggested that age, BMI, TyG index, WBC, NLR, LDH, total PSA, fPSA%, Smoking history, hypertension history, and lesion location are all associated with the risk of malignancy (*P* < 0.05). After incorporating these variables into the multivariable stepwise regression, the independent predictive factors retained in model 1 are: age (OR = 1.0902, 95%CI: 1.0477–1.1368), TyG index(OR = 4.6020, 95%CI: 2.0222–11.6756), NLR (OR = 1.8409, 95% CI: 1.1768–2.9411), free PSA% (OR = 0.9499, 95%CI: 0.9118–0.9887), smoking history (OR = 3.1342, 95%CI: 1.6992–5.8948), hypertension (OR = 2.8802, 95%CI: 1.5792–5.3635) and lesion location (OR = 0.2958, 95%CI: 0.1549-0.5521) (**Table 3**).

**Table 3:** Multivariate logistic regression analyses for predicting prostate cancer (Model 1) and high-grade classification (Model 2).

Model/Variable	OR	Lower CI	Upper CI	P
Model 1				
Age	1.0902	1.0477	1.1368	**<0.001**
BMI	1.0773	0.9939	1.1702	0.073
TyG index	4.6020	2.0222	11.6756	**<0.001**
WBC	1.0241	0.9284	1.1305	0.634
NLR	1.8409	1.1768	2.9411	**0.009**
LDH	1.0103	0.9979	1.0233	0.110
tPSA	1.0740	0.9898	1.1715	0.095
Free PSA%	0.9499	0.9118	0.9887	**0.012**
Smoking history	3.1342	1.6992	5.8948	**<0.001**
Hypertension	2.8802	1.5792	5.3635	**<0.001**
Lesion location	0.2958	0.1549	0.5521	**<0.001**
Model 2				
Age	1.0599	1.0136	1.1120	**0.013**
TyG index	2.6088	1.1000	6.8419	**0.043**
PLT	1.0067	0.9952	1.0186	0.255
NLR	1.2465	0.8205	1.9046	0.303
LDH	1.0166	1.0047	1.0294	**0.008**
Free PSA%	0.9662	0.9238	1.0089	0.124
Smoking history	4.1855	1.8313	10.2068	**0.001**

Variables with P < 0.05 in univariate analysis were entered into multivariate backward stepwise regression using AIC. Bold values indicate statistical significance. OR, odds ratio; CI, confidence interval; BMI, body mass index; TyG, triglyceride-glucose index; WBC, white blood cell; NLR, neutrophil-to-lymphocyte ratio; LDH, lactate dehydrogenase; tPSA, total prostate-specific antigen. Reference categories: smoking history (No), hypertension (No), lesion location (Peripheral Zone).

For the model for distinguishing the pathological grading of malignant prostate lesions (hereinafter referred to as Model 2), through univariate analysis, we found that Age, TyG index, PLT, NLR, LDH, free PSA%, and smoking history were all associated with the risk of high-grade malignancy (*P* < 0.05). In Model 2,we perform a similar multivariate logistic regression analysis, and the variables retained in the model 2 include: age(OR = 1.0599, 95%CI:1.0136-1.1120), TyG index(OR = 2.6088, 95%CI:1.1000-6.8419), LDH(OR = 1.0166, 95%CI:1.0047-1.0294) and smoking history(OR = 4.1855, 95%CI:1.8313-10.2068) (**Table 3**).

### Construction of models

**Figure 1:**
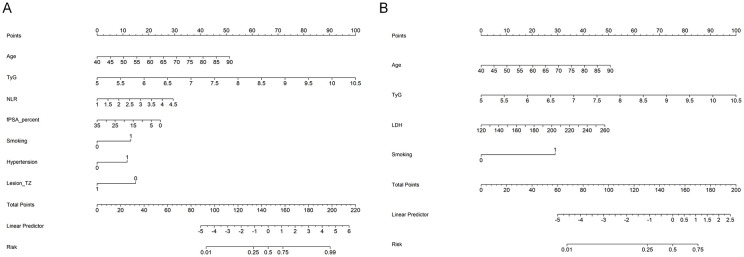
Nomogram for predicting prostate cancer risk (Model 1) and high-grade risk (Model 2). Establishment of two predicting nomograms. **(A)** Model 1 for predicting benign vs. malignant. **(B)** Model 2 for predicting high-grade vs. low-grade among malignant patients.

**Figures 1A, B** respectively show the nomograms of Model 1 (predicting benign and malignant prostate) and Model 2 (predicting high-grade cancer). Each variable is assigned a corresponding score range, so we can directly obtain scores on the corresponding axis based on the values of each variable, calculate the total score, and map it to the prediction probability. For example, a 50 year old patient who does not smoke and does not have hypertension, with a TyG index of 8.1, NLR of 4, and Free PSA% of 20%, calculated a total score of 100 in Model 1, has a probability of developing prostate cancer of approximately 5%. Similarly, a 50 year old non-smoking patient with TyG index=8.1 and LDH = 180U/L calculated a score of 85 in Model 2, corresponding to a probability of only about 6% for developing high-grade prostate cancer.

We presented the multivariate odds ratios and their 95% confidence intervals for both models as forest plots.

**Figure 2:**
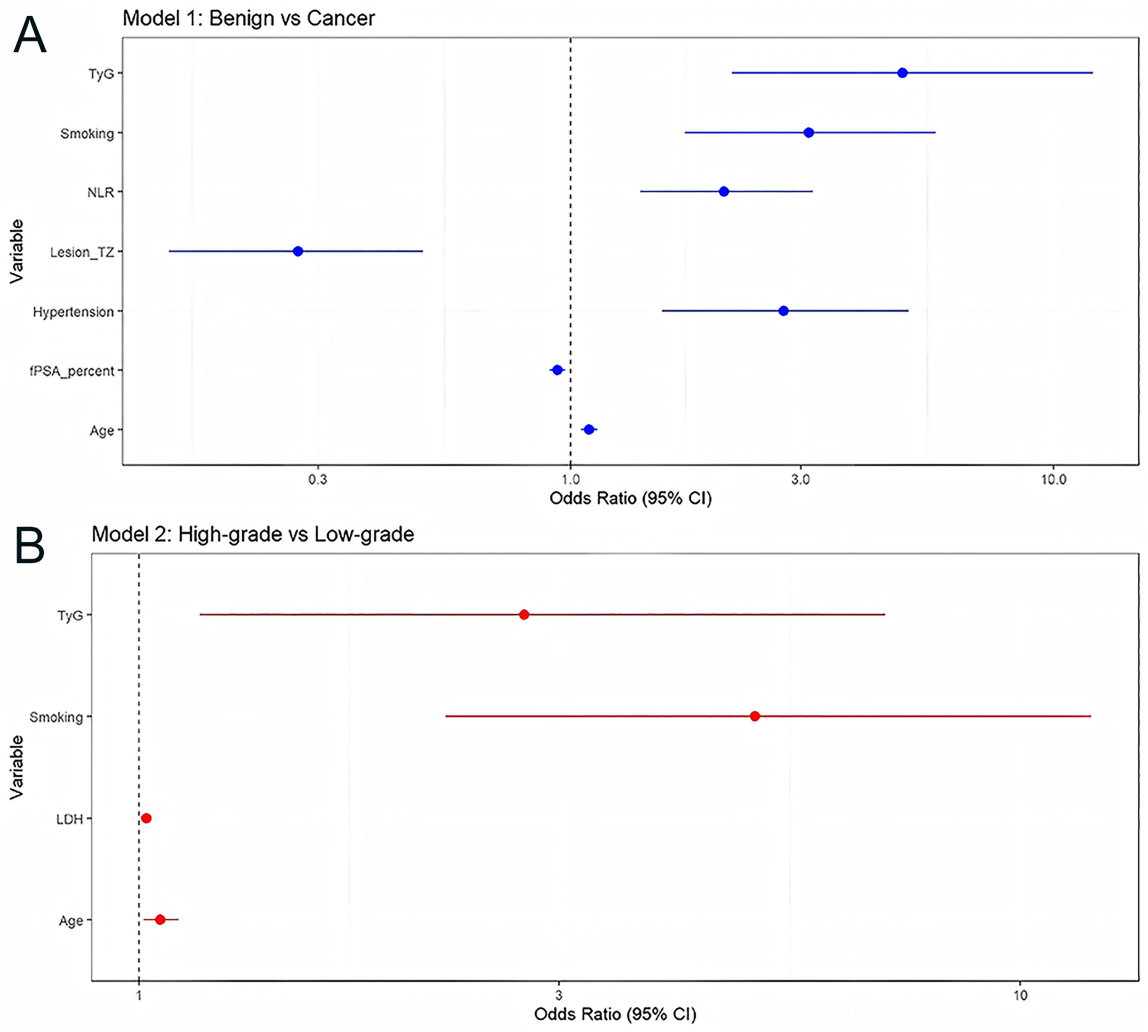
Forest plots of multivariate odds ratios (ORs) with 95% confidence intervals for Models. Forest plots of multivariate odds ratios for Models. **(A)** Model 1 (benign vs. malignant). **(B)** Model 2 (high-grade vs. low-grade). Blue and red squares represent point estimates; horizontal lines indicate 95% CIs. The vertical dashed line at OR = 1 denotes no effect. Variables with OR > 1 are risk factors; OR < 1 are protective factors.

For model 1, TyG, NLR, Hypertension and smoking show strong positive associations with cancer risk, wherever the lesion is located in the transition zone or central zone (compared with peripheral zone) was protective. Unlike model 1, model 2 shared a different pattern: all predictors increased the odds of high grade classification. TyG index, age, LDH, and smoking history are all risk factors, with a higher OR for TyG index and smoking history indicating a stronger risk. The multivariate odds ratios forest plots of two models are shown in **Figure 2**.

### Model performance

**Figure 3:**
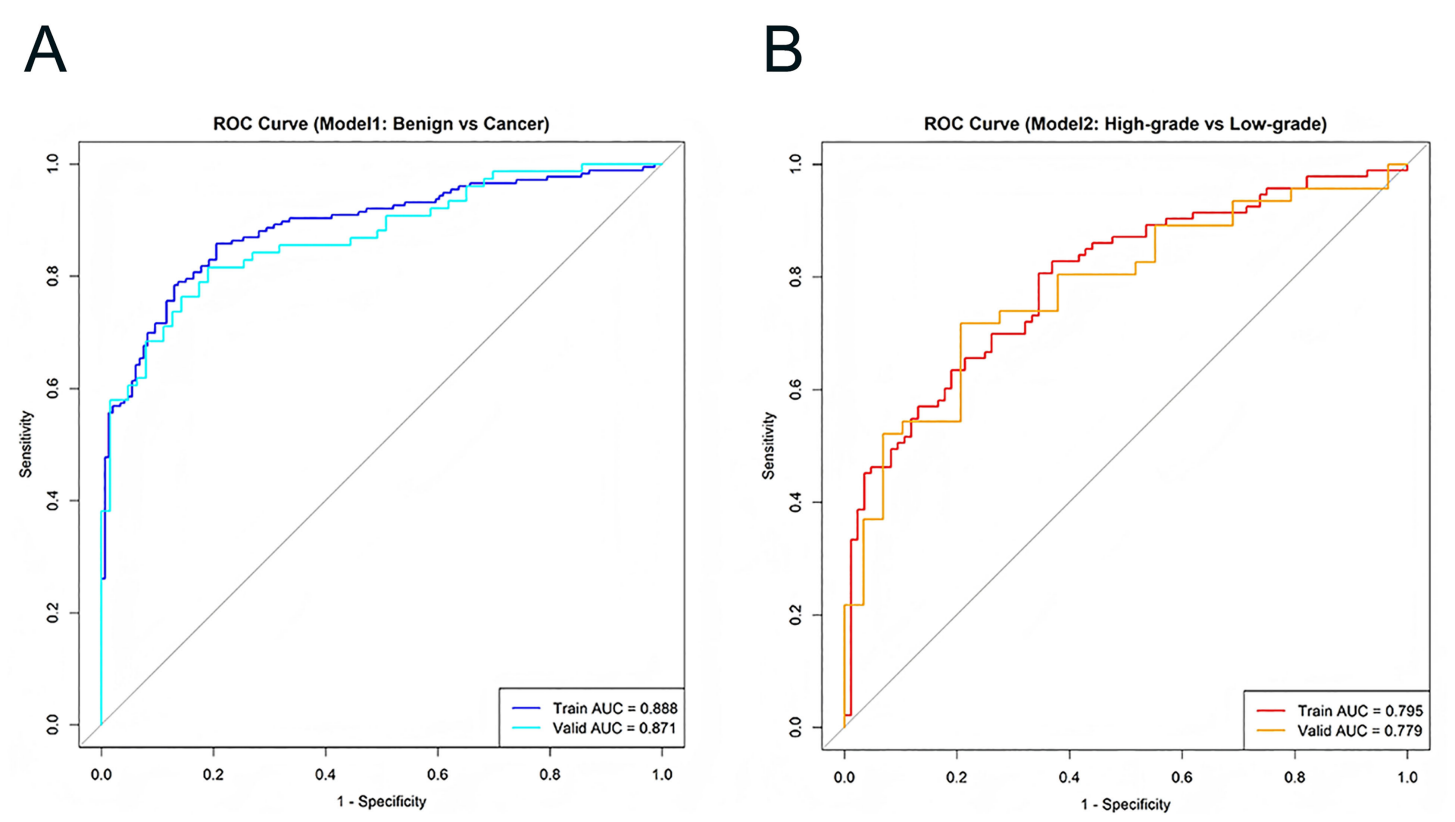
ROC curves of Model 1 and Model 2 in training and validation sets. ROC curves of models. **(A)** ROC curves of Model 1 (benign vs. malignant). **(B)** ROC curves of Model 2 (high-grade vs. low-grade). The diagonal dashed line represents the reference of no discrimination.

To evaluate the performance of the two models, we assessed their discriminative ability in the training set and independent validation set. Model 1 achieved an AUC of 0.888 in the training set and 0.871 in the validation set. Model 2 showed similar discrimination (training AUC = 0.795, validation AUC = 0.779). The ROC curves of the two models are shown in **Figure 3**.

We also calculated the optimal probability thresholds determined by two models based on the Youden index (Youden index=sensitivity+specificity-1). When Model 1 takes a threshold of 0.531 in the validation set, the sensitivity is 0.803 and the specificity is 0.746. This means that at this threshold, the model can correctly identify about 80% of malignant patients, while correctly excluding about 75% of benign patients. When Model 2 takes the optimal threshold of 0.543 in the validation set, the sensitivity is 0.717 and the specificity is 0.621; The model can identify approximately 72% of high-grade cancer patients and correctly exclude approximately 62% of low-grade cancer patients. At the optimal thresholds, Model 1 achieved a PPV of 0.792, NPV of 0.758, and accuracy of 0.777 in the validation set. Model 2 achieved a PPV of 0.750, NPV of 0.581, and accuracy of 0.680. The performance indicators of the two models in the validation set are summarized in **Table 4**. Although the Youden index selected this threshold to maximize the sum of sensitivity and specificity for model evaluation, a lower threshold could be used in clinical practice to achieve higher sensitivity at the expense of lower specificity, which may be more appropriate for cancer screening. The optimal threshold should be chosen based on the intended clinical application and the trade−off between sensitivity and specificity.

**Table 4:** Performance metrics of the prediction models in the validation sets.

Model	AUC (95% CI)	Best threshold	Sensitivity	Specificity	PPV	NPV	Accuracy
Model 1 (Benign vs Cancer)	0.871 (0.813-0.929)	0.531	0.803	0.746	0.792	0.758	0.777
Model 2 (High vs Low grade)	0.779 (0.646-0.867)	0.543	0.717	0.621	0.750	0.581	0.680

AUC, area under the ROC curve.

**Figure 4:**
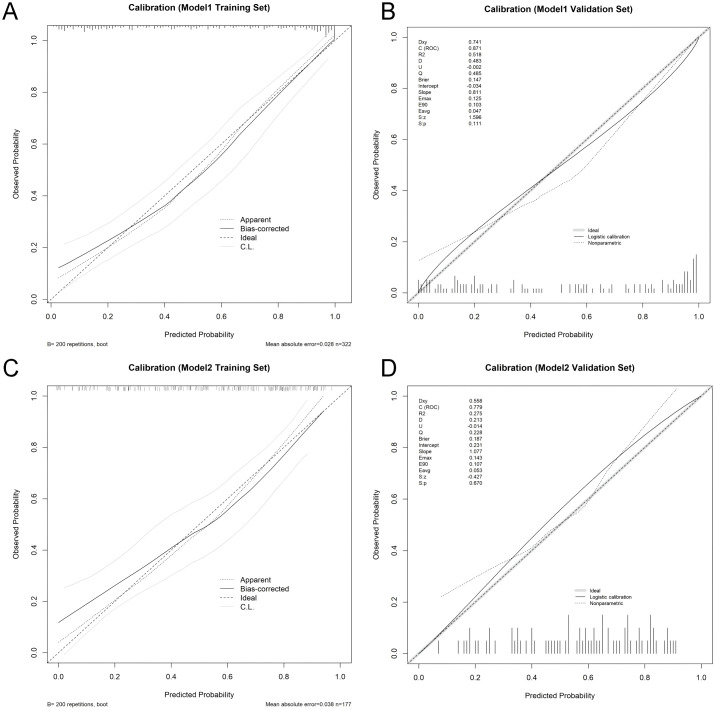
Calibration curves for Model 1 and Model 2 in training and validation sets. Calibration curves for Model 1 and Model 2. **(A)** Model 1 training set. **(B)** Model 1 validation set. **(C)** Model 2 training set. **(D)** Model 2 validation set. The ideal line (dashed) indicates perfect calibration. For training sets, the apparent and bias-corrected curves are shown; for validation sets, the logistic calibration and nonparametric loess curves are presented.

For model 1, in the training set, the Hosmer−Lemeshow test gave a *P* value of 0.1692, meaning no significant difference between predicted probabilities and observed outcomes. The calibration curve (**Figure 4A**) showed that the bootstrap−corrected curve stayed close to the ideal line, with a mean absolute error of 0.028. This suggests the model calibrates well. In the independent validation set, the Hosmer−Lemeshow test also supported good calibration (*P* = 0.378). The calibration curve (**Figure 4B**) showed that both the nonparametric curve and the logistic calibration curve followed the ideal line closely, with a mean absolute error of 0.047. These results confirm that Model 1 has stable calibration ability.

For model 2, the Hosmer−Lemeshow test gave a *P* value of 0.187 in the training set. The bootstrap−corrected calibration curve (**Figure 4C**) showed very small deviations between predicted and observed probabilities, with a mean absolute error of 0.038. In the validation set (**Figure 4D**), Model 2 also showed good calibration performance: the Hosmer−Lemeshow test *P* value was 0.8968 and the mean absolute error was 0.053. These results indicate that Model 2 can provide accurate risk estimates in different datasets.

**Figure 5:**
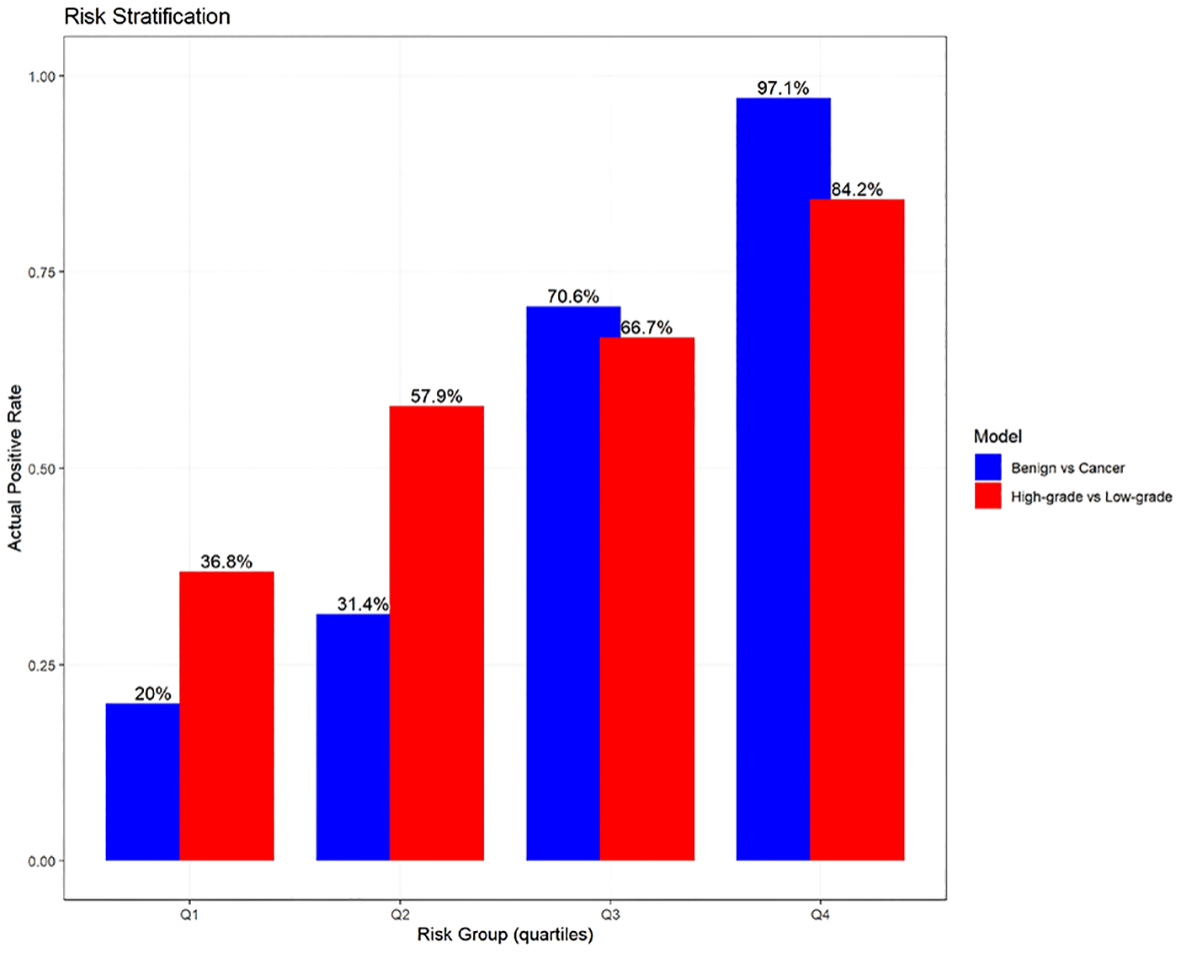
Risk stratification of models based on predicted probability quartiles in validation sets. Patients in the validation set were divided into four groups (Q1–Q4) according to quartiles of the predicted probability. Bars represent the actual positive rate (proportion of malignant or high-grade classification) in each quartile. Error bars indicate 95% confidence intervals.

We further examined how well the models separate different risk levels. Using the validation set, we divided patients into four groups (Q1 to Q4) based on quartiles of the predicted probability from each model. We then calculated the actual proportion of malignant cases (Model 1) or high−grade cases (Model 2) within each quartile. As **Figure 5** shows, the observed event rate rises steadily from Q1 to Q4. For Model 1, the actual malignancy rate increased from 20.0% in the lowest quartile to 97.1% in the highest quartile.

Model 2 also gave a similar graded response for high−grade classification. This monotonic trend indicates that both models effectively discriminate low−risk from high−risk individuals, which supports their potential usefulness in clinical risk triage.

### Decision curve analysis

**Figure 6:**
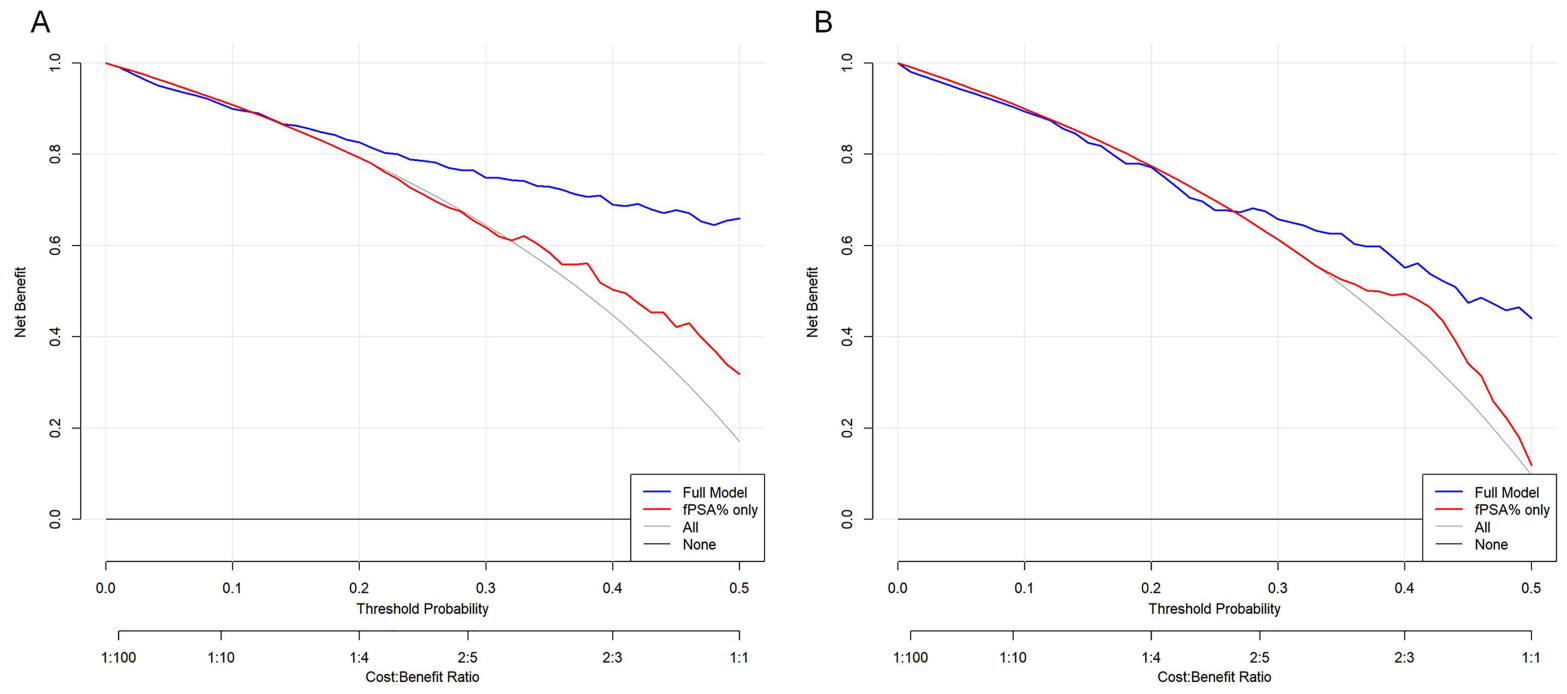
Decision curve analysis of both models. Decision curve analysis of both models compared with fPSA% alone. **(A)** Model 1 vs. fPSA% for predicting prostate cancer. **(B)** Model 2 vs. fPSA% for predicting high-grade classification. The y-axis shows net benefit; the x-axis shows threshold probability. The gray line assumes biopsying all patients; the black line assumes biopsying none. Both models provide higher net benefit than fPSA% alone (red) across a wide range of clinically relevant thresholds.

We performed decision curve analysis to assess the clinical utility of our models. **Figure 6** compares the net benefit of the full model (Model 1 for cancer detection, Model 2 for high−grade classification) against using fPSA% alone and against two extreme strategies (biopsy all patients or biopsy none). For distinguishing between benign and malignant lesions (**Figure 6A**), the full model 1 provides a higher net benefit than fPSA% alone across a wide range of clinically relevant threshold probabilities. Similarly, for predicting ISUP grade of lesions among malignant patients (**Figure 6B**), the full model 2 outperforms fPSA% alone. This indicates that using our model to guide decisions would save more men from unnecessary biopsy while still identifying those with aggressive cancer. DeLong test confirmed that both full models outperformed fPSA% alone (P < 0.05).

The DCA results indicate that our multidimensional models can improve clinical decision-making for diagnosing prostate cancer in males with tPSA>10ng/mL.

## Discussion

In this retrospective study, we developed and validated two prediction models. Model 1 distinguishes benign from malignant prostate lesions, and Model 2 identifies high−grade (ISUP ≥3) cancers among malignant cases. Both models integrate routine clinical and laboratory variables, including age, TyG index, NLR, smoking history, and lesion location. Our models showed good discrimination (validation AUC 0.871 for Model 1, 0.779 for Model 2), satisfactory calibration, and clinically meaningful net benefit across a range of threshold probabilities. The full models outperformed fPSA% alone, which supports the added value of incorporating metabolic and inflammatory markers in the tPSA > 10 population.

As is well known, in the diagnosis of prostate cancer, the tumor marker PSA level is the most commonly used initial screening method, and prostate biopsy is the final diagnostic method (35). In clinical practice, it’s often found that patients have elevated PSA levels, but the biopsy results indicate non tumor status, which increases the patient’s pain and economic costs. Although fPSA% can assist in diagnosis and reduce unnecessary punctures under certain conditions (total PSA 4–10 ng/mL), its reference value decreases when tPSA increases above 10ng/mL. Our results confirm that fPSA% alone gave lower AUC and lower net benefit than our models. Considering the heterogeneity of cancer in different individuals, we believe it is necessary to explore other biomarkers that can accurately diagnose prostate cancer early. Due to the significant difference in prognosis between low-grade and high-grade prostate cancer, if more accurate non-invasive models can be developed to predict the high and low pathological grades of prostate cancer before treatment, it will greatly help improve the overall benefits of comprehensive treatment for prostate cancer.

Some previous studies have shown that insulin resistance is associated with prostate cancer risk (36, 37). The TyG index is a newly developed simple indicator for insulin resistance in recent years. Some research found that a high TyG index was associated with higher cancer risk and worse prognosis (17, 38, 39). These previous research results are consistent with the findings of this study, demonstrating the important value of TyG index in the diagnosis and prognosis evaluation of prostate cancer. However, the difference is that previous studies have mostly considered the TyG index as an important prognostic factor for prostate cancer, mainly because the TyG index reflects the patient’s metabolic level. Abnormal metabolic levels can affect the treatment effectiveness of tumors and the patient’s physical condition. Our research focuses on the possibility that insulin resistance reflected by abnormal TyG index may lead to higher cancer risk and more malignant high-grade tumors. Our research and previous studies have confirmed the close association between various metabolic disorders, including insulin resistance, and prostate cancer from both causal and outcome perspectives. In addition to the TyG index, the other two independent predictive factors that appeared in both models in our study were smoking history and age. Smoking, as a globally recognized risk factor, causes multi-organ damage to health and is closely related to various diseases such as respiratory diseases, cardiovascular diseases, and cancer. Patients with a history of smoking exhibit significantly higher frequency of somatic gene mutations, higher prostate cancer risk and poorer prognosis (40–42). This supports the use of smoking history as one of the evaluation indicators in our model, demonstrating the rationality of the model. Our research also found that smoking rates are higher in malignant patients than in benign patients, and also higher in high-grade than in low-grade patients.

As for age, it has long been regarded as an evaluation indicator for prostate cancer (43–45). It is an undeniable fact that the elderly population has a higher incidence of cancer. Of course, with the popularization of early screening, prostate cancer is showing a trend toward younger patients (46, 47). However, it is still believed that elderly age is one of the characteristics of prostate cancer patients, which is consistent with the findings in this study. Our research also supports this conclusion, showing that malignant patients are older than benign patients, and high-grade patients are older than low-grade patients. In our study, we also found that indicators such as NLR and LDH were identified as independent predictive factors for diagnosing prostate cancer. Inflammation has been widely recognized as one of the hallmarks of tumors, and NLR, as an indicator of inflammation in the body, has also been extensively studied for its association with prostate cancer (30, 48, 49). LDH is a known marker of tumor load and aggression (50, 51). Due to the Warburg effect of tumor cells, they synthesize a large amount of LDH, so an increase in LDH often indicates a high tumor burden or strong tumor invasiveness (52–54).

Several risk stratification tools for prostate cancer currently exist, as summarized in a recent review (55). These include the D’Amico risk groups, CAPRA score, MSKCC nomogram, and the PREDICT Prostate model, which primarily rely on PSA, Gleason score, and clinical T−stage. Some tools also incorporate comorbidities or the percentage of positive biopsies. However, many of these tools were developed in selected cohorts treated in different time frames and may not fully account for modern diagnostic workups or the metabolic and inflammatory landscape of prostate cancer. More recently, researchers have integrated advanced imaging or immune markers to improve risk stratification. For example, one study developed a nomogram combining deep learning, PI−RADS scoring, and clinical variables to identify clinically significant prostate cancer on MRI, achieving an AUC of 0.81 (56). Another study constructed a clinical machine learning nomogram using functional subsets of peripheral lymphocytes to predict prostate cancer risk, with an AUC of 0.864 (57). Although these models show promising performance, they require specialized imaging equipment or complex immunological assays, limiting their accessibility in primary care or resource−limited settings. Our models differ by integrating routine metabolic and inflammatory markers that reflect the host’s systemic metabolic state, which has been increasingly recognized as a contributor to tumor aggressiveness. Moreover, our models require only standard blood tests and basic demographic information, making them easily deployable in settings where MRI or genomic panels are unavailable.

In fact, there are many risk assessment tools for prostate cancer now, such as PCA3 levels, MRI, or gene panels (58–60). These tools have also demonstrated good predictive ability and are increasingly being used in clinical practice. Unlike these prediction tools that need costly or less accessible tests, our models only need routine blood tests and basic patient’s demographic information. That makes them easy to use in primary care or places with few resources. Nomograms we developed can help urologists and patients talk about whether to do a biopsy or watchful waiting, especially when tPSA > 10. With Model 1, a patient who has a low predicted risk of cancer could safely skip an immediate biopsy. This reduces unnecessary procedures and patient anxiety. For patients who diagnosed with cancer, Model 2 gives a risk estimate for high−grade disease, which provides important reference information for individualized treatment decisions of patients in the future. What we want to emphasize is that the purpose of the nomograms we constructed is to supplement existing diagnostic methods, not replace them.

These nomograms have several limitations. First, this is a single−center retrospective study, which may introduce selection bias. Second, our nomograms did not include MRI or new biomarkers (such as PCA3). Adding them could improve performance, but they cost more and are less available. Third, the TyG index and LDH can be affected by other metabolic diseases or drugs, and we did not fully control for that. Additionally, although we allowed a one-month window for pre-biopsy laboratory tests, most patients underwent testing within one to two weeks, and we excluded acute prostatitis to minimize PSA fluctuation. Thus, regression dilution bias is unlikely to have substantively affected our conclusions. Fifth, model 2 showed moderate discrimination (AUC 0.779) and acceptable calibration, its relatively low specificity (62.1%) suggests that it should be used in conjunction with other clinical information rather than as a standalone decision tool for high-grade disease. Finally, we used the dataset from a single medical center for both training and internal validation. More external validation is needed.

## Conclusion

For men presenting with tPSA>10 ng/mL, the two nomograms we developed and validated, which integrate demographic, metabolic, inflammatory, and routine tumor markers, can assist in risk stratification for both prostate cancer presence and high−grade classification. These models outperform the conventional fPSA% alone and offer good calibration and net clinical benefit. Our tools can help reduce unnecessary biopsies and guide personalized treatment decisions in the tPSA > 10 population.

## Data Availability

The raw data supporting the conclusions of this article will be made available by the authors, without undue reservation.
