# Supplementary material for: Development and validation of multidimensional nomograms for predicting prostate cancer risk: a retrospective study
Source: Front Oncol. 2026 Jun 30;16:1883224. doi: 10.3389/fonc.2026.1883224 (PMC13364587; doi:10.3389/fonc.2026.1883224)
Supplement: Supplementary file 1 [file Table1.docx]

**Supplementary Table 1 Variance inflation factor (VIF) of all variables**

| Variable | VIF |
| --- | --- |
| Age | 1.0557 |
| BMI | 1.0430 |
| FPG | 22.1515 |
| TyG | 2.0680 |
| TC | 28.6652 |
| LDL | 26.8611 |
| HDL | 4.1874 |
| RBC | 78.2941 |
| Hb | 78.1901 |
| PLT | 1.0479 |
| WBC | 1.2520 |
| NEUT | 15.1516 |
| LYM | 14.1516 |
| NLR | 1.2507 |
| LDH | 1.1821 |
| ALT | 79.9156 |
| AST | 100.4755 |
| ALP | 83.9454 |
| tPSA | 1.2684 |
| fPSA | 11.1562 |
| fPSA_percent | 1.3300 |
| Smoking | 1.1667 |
| Hypertension | 1.0619 |
| Lesion_TZ | 1.3334 |
| Lesion_CZ | 1.1746 |

Abbreviation：BMI: Body Mass Index; FPG: Fasting Plasma Glucose; TyG: Triglyceride-Glucose Index; TC: Total Cholesterol; LDL: Low-Density Lipoprotein; HDL: High-Density Lipoprotein; RBC: Red Blood Cell Count; Hb: Hemoglobin; PLT: Platelets; WBC: White Blood Cell Count; NEUT: Neutrophils; LYM: Lymphocytes; NLR: Neutrophil-to-Lymphocyte Ratio; LDH: Lactate Dehydrogenase; ALT: Alanine Aminotransferase; AST: Aspartate Aminotransferase; ALP: Alkaline Phosphatase; tPSA: Total Prostate-Specific Antigen; fPSA: Free Prostate-Specific Antigen; fPSA_percent: Free-to-Total PSA Ratio; Smoking: Smoking status; Hypertension: Hypertension status; Lesion_TZ: Lesion in Transition Zone; Lesion_CZ: Lesion in Central Zone
