# Supplementary material for: Development and validation of multidimensional nomograms for predicting prostate cancer risk: a retrospective study
Source: Front Oncol. 2026 Jun 30;16:1883224. doi: 10.3389/fonc.2026.1883224 (PMC13364587; doi:10.3389/fonc.2026.1883224)
Supplement: Supplementary file 2 [file Table2.docx]

**Supplementary Table 2 Univariate logistic regression analyses for predicting prostate cancer (Model 1) and high-grade classification (Model 2)**

| Model/Variable | OR | Lower CI | Upper CI | *P* |
| --- | --- | --- | --- | --- |
| Model 1 |  |  |  |  |
| Age | 1.0957 | 1.0614 | 1.1335 | **＜0.001** |
| BMI | 1.0800 | 1.0179 | 1.1475 | **0.012** |
| TyG | 4.1911 | 2.0942 | 8.5824 | **＜0.001** |
| HDL | 1.3697 | 0.6341 | 2.9717 | 0.424 |
| PLT | 1.0058 | 0.9985 | 1.0133 | 0.119 |
| WBC | 1.0630 | 0.9976 | 1.1340 | **0.061** |
| NLR | 2.5368 | 1.8733 | 3.5010 | **＜0.001** |
| LDH | 1.0161 | 1.0073 | 1.0254 | **＜0.001** |
| tPSA | 1.1470 | 1.0840 | 1.2194 | **＜0.001** |
| Free PSA% | 0.9198 | 0.8928 | 0.9463 | **＜0.001** |
| Smoking | 3.8437 | 2.4095 | 6.2074 | **＜0.001** |
| Hypertension | 2.4917 | 1.5913 | 3.9339 | **＜0.001** |
| Lesion location | 0.2184 | 0.1354 | 0.3476 | **＜0.001** |
| Model 2 |  |  |  |  |
| Age | 1.0710 | 1.0280 | 1.1199 | **0.002** |
| BMI | 1.0229 | 0.9460 | 1.1070 | 0.570 |
| TyG | 3.1327 | 1.4278 | 7.4926 | **0.007** |
| HDL | 0.6670 | 0.2356 | 1.8703 | 0.442 |
| PLT | 1.0129 | 1.0029 | 1.0235 | **0.013** |
| WBC | 1.0268 | 0.9425 | 1.1196 | 0.546 |
| NLR | 1.5540 | 1.0949 | 2.2321 | **0.015** |
| LDH | 1.0174 | 1.0070 | 1.0284 | **0.001** |
| tPSA | 1.0178 | 0.9593 | 1.0809 | 0.561 |
| Free PSA% | 0.9589 | 0.9233 | 0.9946 | **0.026** |
| Smoking | 4.5874 | 2.1843 | 10.2675 | **＜0.001** |
| Hypertension | 1.5065 | 0.8286 | 2.7545 | 0.180 |
| Lesion location | 0.7761 | 0.4044 | 1.4841 | 0.443 |

Note: Variables with P < 0.05 in univariate analysis were entered into multivariate backward stepwise regression using AIC. Abbreviations: OR, odds ratio; CI, confidence interval; BMI, body mass index; TyG, triglyceride-glucose index; WBC, white blood cell; NLR, neutrophil-to-lymphocyte ratio; LDH, lactate dehydrogenase; tPSA, total prostate-specific antigen. Reference categories: smoking history (No), hypertension (No), lesion location (Peripheral Zone).
